# Single-stage debridement with implantation of antibiotic-loaded calcium sulphate in 34 cases of localized calcaneal osteomyelitis

**DOI:** 10.1080/17453674.2020.1745423

**Published:** 2020-04-02

**Authors:** Nan Jiang, Xing-qi Zhao, Lei Wang, Qing-rong Lin, Yan-jun Hu, Bin Yu

**Affiliations:** a Department of Orthopaedics & Traumatology, Nanfang Hospital, Southern Medical University, Guangzhou;; b Guangdong Provincial Key Laboratory of Bone & Cartilage Regenerative Medicine, Nanfang Hospital, Southern Medical University, Guangzhou, P R China

## Abstract

Background and purpose — The successful eradication of calcaneus infection with limb salvage remains a challenge. We describe the outcomes of cortical bone windowing followed by eggshell-like debridement and implantation of antibiotic-loaded calcium sulphate (CS) for localized (Cierny–Mader type III) calcaneal osteomyelitis (CO).

Patients and methods — We report a retrospective study of 34 patients. Infection followed trauma or orthopedic surgery in 30 patients and hematogenous spread in 4 patients. 31 patients had a sinus tract, accompanied by a soft tissue defect in 3 patients. All patients received cortical bone windowing, debridement, multiple sampling, local implantation of vancomycin- and gentamicin-loaded CS, skin closure or flap coverage, and culture-specific systematic antibiotic treatment in a single-stage procedure. Patients were followed up for a median of 26 months.

Results — Infection was eradicated in 29 patients after the single-stage surgery, and all of the 5 recurrent infections were cleared by repeated surgery without amputation. Other adverse events included 11 patients with aseptic wound leakage and 1 unrelated death. Compared with those before surgery, the median postoperative scores of the American Orthopaedic Foot & Ankle Society (AOFAS) ankle hindfoot scale (65 vs. 86 vs. 89) and the visual analog scale (VAS) for pain (6 vs. 3 vs. 1) improved at the 1-year and 2-year follow-up.

Interpretation — This single-stage protocol, cortical bone windowing, and eggshell-like debridement combined with local implantation of antibiotic-loaded CS is effective in treating type III CO. However, the incidence of aseptic wound leakage is high.

Calcaneal osteomyelitis (CO) is an uncommon condition, which usually occurs following trauma, orthopedic surgery, diabetic ulcers, and hematogenous spread (Fukuda et al. 2010, Mooney et al. 2017). Infrequent causes are iatrogenic steroid injection and acupuncture (Waibel et al. 2019). The primary goals of CO management are eradicating infection, adequate and durable soft tissue coverage, and maximal maintenance of the foot function (Merlet et al. 2014). The successful treatment of CO with limb salvage remains a challenge, primarily being attributable to the unique anatomical structure and function of the calcaneus, with limited surrounding soft tissue and blood supply. Clinical efficacy remains unsatisfying, with high risks of infection relapse and amputation (Merlet et al. 2014, Sabater-Martos et al. 2019, Waibel et al. 2019).

Calcium sulphate (CS), a novel local antibiotic vehicle, has been widely used for the treatment of chronic osteomyelitis with satisfying outcomes (Gauland 2011, Ferguson et al. 2014, Andreacchio et al. 2019). Compared with polymethylmethacrylate (PMMA), CS can carry a wider range of antibiotics and is completely biodegradable, thus not requiring second surgery for removal (Inzana et al. 2016). Although previous studies had reported local antibiotic-loaded CS implantation for CO treatment, their strategies differed, including a 2-stage surgery of debridement followed by autologous bone graft (Papagelopoulos et al. 2006), the Silo technique with CS/hydroxyapatite (Drampalos et al. 2018), and even calcanectomy (Walsh and Yates 2013). Nonetheless, the clinical experience of bone-preserving strategy for CO treatment remains limited.

To describe the extent of the inflammatory process and determine treatment strategy, Cierny–Mader (C–M) classification (Cierny and Mader 2003) has been proposed, which consists of anatomic type (I: medullary, II: superficial, III: localized, and IV: diffuse) and physiologic class (host A: good immune system and delivery, host B: compromised locally (BL) or systemically (BS) or both (BLS), and host C: not a surgical candidate due to poor systemic condition and prognosis).

Here, we present a series of patients with C–M type III CO treated with a bone-preserving surgical protocol, cortical bone windowing, eggshell-like debridement, and local antibiotic-loaded CS implantation in a 1-stage surgery.

## Patients and methods

### Study design and inclusion and exclusion criteria

This retrospective study was conducted in Nanfang Hospital of Southern Medical University, a tertiary medical center in Southern China. Included patients were those presenting with type III CO of the C–M anatomy classification, following injury, orthopedic surgery, or hematogenous spread, and having inflammatory symptoms for over 10 weeks (Metsemakers et al. 2018a). Type III CO refers to a local infection of the cortical and cancellous bone of the calcaneus, which does not affect the stability of the bone. 2 experienced surgeons made the C–M classification for each included patient independently and disputes were resolved by a third assessor.

The establishment of CO diagnosis is based on at least 1 of the following confirmatory criteria (Metsemakers et al. 2018b): supportive histology, microbiological cultures from at least two suspected sites revealing the same pathogen, a definite sinus tract connecting directly the bone or the implant, or wound purulent drainage, or intraoperative pus. The exclusion criteria were diabetic foot-associated CO, patients with renal failure, calcium metabolism disorders, and a known allergy to CS, vancomycin, or gentamicin. In addition, patients who refused to receive this protocol were also excluded.

Electronic medical records of 59 consecutive patients with chronic CO were screened between January 2013 and December 2017. However, 25 patients were excluded for the following reasons: 15 patients with type II, 6 with type IV, and 4 with type III, including 2 with diabetic foot and 2 with hypercalcemia.

The remaining 34 patients (27 males) involving 34 limbs were included. The mean age at diagnosis was 41 years (3–67). The median follow-up time was 26 months (12–68).

### Preoperative management

After admission, patients underwent routine physical examinations, imaging, and laboratory tests. Microbiology samples from the sinus tract were not taken. Antibiotics were stopped for at least 2 weeks prior to surgery.

### Surgical management

After anesthesia, a tourniquet was routinely placed at the upper thigh. The surgical approach was selected based on the infection location and range, as suggested by the sinus tract location and/or radiographs/MRI scans. The sinus tract was first excised, then necrotic and devascularized soft tissues surrounding the infected calcaneus were debrided. Thereafter, the devitalized cortical bone was windowed and removed using an osteotome, to expose the calcaneus cancellous bone. A curette was used to remove the infected cancellous bone and its surrounding osseous tissues. However, the “calcaneus shape” was carefully protected during debridement and intraoperative radiographs were taken when necessary. After multiple samples were collected and sent for culture and histology, patients received empirical intravenous antibiotics (cephalosporins or alternatively clindamycin). Finally, the infected cancellous and surrounding bone were removed, leaving a cavity in the residual calcaneus as an eggshell. Subsequently, the cavity was soaked in 0.05% aqueous chlorhexidine solution 3 times for 5 minutes, followed by pulsed irrigation of 2–4 L of sterile saline solution. The cavity was dried and packed with gauze.

After changing the gloves and drapes, the antibiotic-loaded CS was made with a mixture of vancomycin, gentamicin, and CS powder (Stimulan Rapid Cure; Biocomposites Ltd, ­Staffordshire, UK). The mixture ratio was 500 mg vancomycin with 5 mL CS dissolved in 2 mL/80 mg gentamicin with an additional 0.6 mL of sterile water. After shaping and solidification, the antibiotic-loaded CS block was filled into the cavity and the surrounding soft tissues. Primary skin closure was either achieved directly or the wound was covered by a local sural neurovascular flap or using the skin-stretching technique (Figure).

### Postoperative management

After surgery, all of the patients were immobilized in a brace until the wound healed. During this period, patients received empirical antibiotics intravenously (cephalosporins or alternatively clindamycin), multimodal analgesia, and supportive treatment. Subsequent antibiotic selections were based on results of cultures and drug sensitivity outcomes of the samples taken intraoperatively. If the culture outcomes were negative, cephalosporins or clindamycin were continued. All patients received antibiotics intravenously for 2 weeks and orally for an additional 4 weeks. Rifampin and/or quinolones were added in patients suspected of having staphylococci and/or gram-negative bacteria-associated biofilm infections. Wound dressings were changed every 2–3 days. For patients who developed continuous drainage, the wound status was observed closely, serological levels of white blood cell count (WBC), erythrocyte sedimentation rate (ESR), and C-reactive protein (CRP) were monitored, and multiple samples were cultured to exclude infection relapse. Patients received functional training with supervision. Partial weight-bearing was allowed after complete wound healing, and full weight-bearing was usually allowed 6–8 weeks after surgery.

### Outcome parameters

Outcome parameters included the infection eradication rate after the 1-stage procedure at 1-year follow-up, adverse events, the American Orthopaedic Foot & Ankle Society (AOFAS) ankle–hindfoot scale score, and the visual analog scale (VAS) for pain score (scale from 0 to 10) before surgery, at 1 year, and if possible at 2 years postoperatively.

### Statistics

Descriptive statistics were conducted using the Statistical Product and Service Solutions (SPSS) 19.0 software (SPSS Inc., Chicago, IL, USA). Distributions of continuous variables were evaluated for normality using the Kolmogorov–Smirnov test initially. Then, data were expressed as mean (SD) and median (range) for normally and abnormally distributed variables, respectively.

### Ethics, funding, and potential conflicts of interest

This study was approved by the Medical Ethics Committee of Nanfang Hospital, Southern Medical University. Informed consent from all included patients or their legal guardian was waived due to the retrospective design. Research was funded by a grant from the National Natural Science Foundation of China (grant number: 81802182). There is no conflict of interest to declare.

**Figure:**
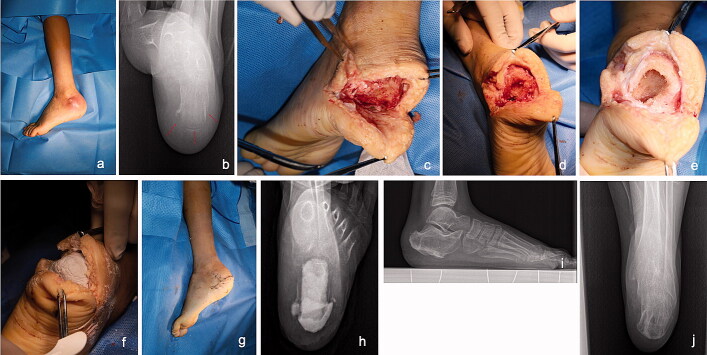


## Results

### Patients

According to the C–M host classification, 3 patients were type A and 31 were type B, including 26 patients as type BL, 1 as type BS, and the remaining 4 as type BLS hosts. 31 patients had at least 1 sinus tract, including 3 patients with accompanying soft tissue defect.

There were 21 and 13 patients with CO on the left and right side of the body, respectively. 30 patients had posttraumatic osteomyelitis, whereas 4 had CO following hematogenous spread. 20 patients suffered from open fractures and 11 patients had infection after fracture fixation. Falling from a height was the most frequent injury type (10 patients), followed by a sharp puncture injury (7 patients) (Table 1).

**Table 1.** Clinical characteristics of the included patients

No.	Sex/age	Cause	Side	Culture outcome	Local comorbidity
1	M/38	Iatrogenic factor	R	Negative	Sinus tract
2	M/60	Iatrogenic factor	L	*Stenotrophomonas maltophilia*	Sinus tract
3	M/29	Falling injury	R	*Pseudomonas aeruginosa*	Sinus tract
4	F/41	Sharp puncture	L	Negative	Sinus tract
5	M/50	Crushing injury	L	*Staphylococcus aureus*	Sinus tract
6	M/64	Falling from a height	L	Polymicrobial	Sinus tract ^a^
7	F/51	Falling from a height	L	Negative	Sinus tract
8	M/52	Traffic accident	R	Negative	Sinus tract
9	M/10	Sharp puncture	L	*Pseudomonas aeruginosa*	Sinus tract
10	M/67	Crushing injury	R	Negative	Sinus tract
11	M/63	Sharp puncture	L	*Pseudomonas aeruginosa*	No
12	M/45	Falling from a height	L	*Escherichia coli*	Sinus tract
13	F/67	Sharp puncture	R	Negative	No
14	M/53	Hematogenous spread	R	Negative	Sinus tract
15	M/54	Sharp puncture	L	Negative	Sinus tract
16	F/51	Falling from a height	L	Negative	Sinus tract
17	M/56	Frostbite injury	R	Negative	Sinus tract
18	M/30	Falling from a height	L	Negative	Sinus tract ^a^
19	M/20	Sharp puncture	L	Negative	Sinus tract
20	M/10	Hematogenous spread	L	*Enterobacter cloacae*	No
21	M/33	Traffic accident	R	*Proteus mirabilis*	Sinus tract
22	M/12	Sharp puncture	L	*Pseudomonas aeruginosa*	Sinus tract
23	M/25	Traffic accident	L	Negative	Sinus tract
24	F/60	Falling from a height	L	*Staphylococcus aureus*	Sinus tract
25	M/36	Sprain injury	R	*Enterobacter cloacae*	Sinus tract
26	F/59	Hematogenous spread	L	Polymicrobial	Sinus tract
27	F/65	Falling from a height	L	Negative	Sinus tract
28	M/3	Iatrogenic factor	R	Negative	Sinus tract
29	M/22	Falling from a height	L	Negative	Sinus tract
30	M/29	Falling from a height	L	Negative	Sinus tract
31	M/43	Falling injury	L	Negative	Sinus tract
32	M/18	Hematogenous spread	R	Negative	Sinus tract
33	M/48	Falling injury	R	Negative	Sinus tract
34	M/46	Falling from a height	R	*Pseudomonas aeruginosa*	Sinus tract ^a^

**^a^**Sinus tract + soft tissue defect

#### Inflammatory biomarkers and microbiology

The median serological levels of WBC, ESR, and CRP were 7.2 (4.4–14) × 10^9^/L, 16 (2–140) mm/h, and 4.7 (0.2–228) mg/L, with abnormal levels in 5/34, 16/33, and 14/31 patients, respectively.

14 of 34 patients had a positive pathogen culture outcome, with 12 patients having monomicrobial and 2 having polymicrobial infections. *Pseudomonas aeruginosa* (5 cases) was the most frequently detected bacteria, followed by *Enterobacter cloacae* (2 cases) and *Staphylococcus aureus* (2 cases) (Table 1). All of the cultured microorganisms were sensitive to vancomycin and/or gentamycin.

#### Infection eradication rate and adverse events

The median CS volume implanted was 20 (5–40) mL. Skin closure was primarily achieved in 31 patients, with 2 covered by a local sural neurovascular flap and 1 by the skin-stretching technique.

Infection was successfully eradicated in 29 patients following the 1-stage procedure at the 1-year follow-up. All 5 patients with recurrent infections received secondary debridement surgery, with 2 patients receiving wound irrigation, 2 receiving antibiotic-loaded CS implantation, and the remaining patient receiving local flap coverage. All of these 5 patients had recovered well at the median follow-up of 14 months (14–26).

11 patients had aseptic wound leakage, with a median duration of 31 days (16–52). Of these, 10 were managed with wound dressing and 1 underwent a secondary debridement surgery to remove the residual CS; all of these patients recovered well. In addition, 1 patient died of a cause unrelated to CO or the surgery, as his wound and foot function had recovered well at the 1-year follow-up. Moreover, none of the patients had limb amputation or secondary fractures during follow-up (Table 2).

**Table 2.** Clinical efficacy of the included patients

			AOFAS score			VAS pain score		
No.	Sex/age	Adverse events	Before surgery	1 year after surgery	2 years after surgery	Before surgery	1 year after surgery	2 years After surgery
1	M/38	Aseptic wound leakage	72	87	89	6	2	0
2	M/60	Nil	65	87	90	5	3	0
3	M/29	Nil	39	84	90	7	3	2
4	F/41	Nil	43	80	80	7	3	1
5	M/50	Aseptic wound leakage	65	87	89	6	3	2
6	M/64	Aseptic wound leakage	68	90		6	3	
7	F/51	Infection relapse	58	86		6	3	
8	M/52	Aseptic wound leakage	65	82	84	6	4	1
9	M/10	Aseptic wound leakage	68	90	90	6	2	1
10	M/67	Aseptic wound leakage	62	86		6	2	
11	M/63	Unrelated death	72	86		5	2	
12	M/45	Nil	72	89	90	6	3	1
13	F/67	Nil	82	86	87	6	4	2
14	M/53	Nil	61	84	87	6	2	1
15	M/54	Aseptic wound leakage	61	87	87	6	2	0
16	F/51	Nil	49	90	90	7	2	0
17	M/56	Nil	68	87	87	6	4	1
18	M/30	Aseptic wound leakage	65	86		6	3	
19	M/20	Nil	79	90	90	4	3	0
20	M/10	Nil	51	90	90	6	2	0
21	M/33	Nil	46	84		7	3	
22	M/12	Nil	73	90	90	5	2	0
23	M/25	Infection relapse	72	84	84	7	3	2
24	F/60	Infection relapse	65	85	90	5	4	2
25	M/36	Nil	81	90		4	2	
26	F/59	Nil	72	84		6	2	
27	F/65	Infection relapse	45	75		6	4	
28	M/3	Nil	71	90		6	2	
29	M/22	Aseptic wound leakage	52	78	78	6	4	2
30	M/29	Nil	56	84		6	2	
31	M/43	Infection relapse	68	86		6	3	
32	M/18	Aseptic wound leakage	75	90		4	2	
33	M/48	Nil	58	84		5	3	
34	M/46	Aseptic wound leakage	58	89		6	2	

AOFAS: American Orthopaedic Foot & Ankle Society; VAS: visual analog scale.

#### AOFAS ankle–hindfoot scale score before and after surgery

The median AOFAS ankle–hindfoot scale score of the 34 patients was 65 (39–82) before surgery, which increased to 86 (75–90) at the 1-year follow-up, and to 89 (78–90) at the 2-year follow-up after surgery (Table 2).

#### VAS scores before and after surgery

The median VAS score for pain was 6 (4–7) before surgery, which decreased to 3 (2–4) at the 1-year follow-up, and to 1 (0–2) at the 2-year follow-up postoperatively (Table 2).

## Discussion

Successful treatment of CO with limb preservation remains a challenge for clinicians, not only because of the unique structure and function of the calcaneus, but also owing to the limited surrounding soft tissue coverage and poor blood supply. Previous studies had indicated that the risk of secondary below-knee amputation following limb-saving surgery in CO patients ranged from 4% to 20% (Baumhauer et al. 1998, Schade 2012, Van Riet et al. 2012, Walsh and Yates 2013, Babiak et al. 2016). Even a recent study (Waibel et al. 2019) showed that 50% of the CO patients had to undergo secondary below-knee amputation even if they had already received total calcanectomy. The optimal surgical strategy for CO remains controversial (Sabater-Martos et al. 2019). We have reported the outcomes of the cortical bone windowing followed by eggshell-like debridement and local vancomycin- and gentamicin-loaded CS for the treatment of patients with type III CO. Our findings can be summarized under the following 4 aspects.

First, the infection eradication rate (29/34) after the single-stage surgery was similar to those of previous studies (Ferguson et al. 2014, Luo et al. 2016, Ferguson et al. 2017). Whether bone infection relapses or not after treatment is influenced by multiple factors, such as surgical and antibiotic strategies, pathogen species and virulence, and host immune status. Among these factors, debridement plays a vital role, which should not be limited by concerns of repairing osseous/soft tissue defects (Metsemakers et al. 2018a). The goal of infection excision is to remove all the devitalized tissues, leaving behind healthy vascularized bone and managing the subsequent dead space to prevent re-accumulation of hematoma that may become re-infected. It is reasonable to understand that the protocols for CO treatment include partial and total calcanectomy, or even below-knee amputation (Waibel et al. 2019). Although infection can be eradicated following such radical surgeries, the foot function may be more or less impaired. The outcomes of a recent study (Waibel et al. 2019) including 50 patients indicated that the secondary re-amputation proportions of the CO patients following partial/total calcanectomy and below-knee amputation were 8/28, 2/4, and 1/18, respectively, implying the potential problems of such surgical strategies. Considering the importance of the weight-bearing function of the calcaneus, we tried to maintain the integrity of the external cortical shell of the calcaneus during debridement, similar to the approach of a previous report (Papagelopoulos et al. 2006). However, they used a PMMA cement carrier and, thus, performed secondary autologous bone graft surgery, whereas in our study we only performed 1-stage surgery, facilitated by CS being totally biodegradable and not requiring a second surgery. It should be noted that, in this study, we included only non-diabetic foot patients, which may be the primary reason for our much lower amputation rate compared with those of previous studies.

Second, incidence of the adverse events apart from infection relapse was 12/34, with aseptic wound leakage being the most frequently observed. Previous studies reported that the proportion of aseptic wound leakage ranged from 6/100 (McNally et al. 2016) to 7/21 (Humm et al. 2014), showing an average incidence of 16% (Ferguson et al. 2017), whereas, it was 11/34 in our patients, higher than most of the previous studies. The difference may be primarily attributed to 2 reasons. 1 is owing to the limited soft tissue coverage as well as blood supply to the calcaneus physiologically. In the case of bone infection, the local soft tissue might worsen, which aggravates the blood circulation locally. Thus, there is an increased risk of wound problem. The other reason may be related to the limited sample size of our study. To evaluate more accurately whether wound leakage risk after CS local use in the calcaneus is higher than with CS implanted in other bones, studies with larger sample sizes are warranted. In addition to the wound leakage, local CS implantation may also bring other problems, such as heterotopic ossification (HO) (Kallala et al. 2018) and even hypercalcemia (Kallala and Haddad 2015), which appears to be more frequently found in patients with prosthetic joint infections.

Third, we found that, compared with those before surgery, the median AOFAS ankle–hindfoot scale and VAS scores for pain improved at the 1-year and 2-year follow-ups, demonstrating the restoration of foot function and the alleviation of pain. Such changes might be associated with absolute infection eradication as well as preservation of calcaneus integrity. In a recent study, Babiak et al. (2016) compared the efficacy between bone-preserving strategies (debridement or drilling and collagen-gentamicin sponge) and radical surgical protocols (partial or total calcanectomy) for CO treatment. Outcomes revealed that the proportion of patients who were ambulatory before therapy retained their walking ability after the bone-preserving surgeries was higher than that of the patients receiving radical surgical interventions, suggesting that, on the premise of infection eradication, calcaneus integrity should be preserved as much as possible.

Fourth, we found several unique characteristics of our patients. Initially, the proportion of patients with a sinus tract was much higher, demonstrating compromised local status in most CO patients. Additionally, even sharp puncture injury and iatrogenic injection or acupuncture may lead to calcaneal infection, although such causes are rare (Waibel et al. 2019). Despite the 2-week non-administration of antibiotics and the high number of patients with a drainage sinus tract, the positivity rate of the culture in our study was low, which may be associated with the suboptimal culture conditions. The most frequently detected pathogen in this cohort was *Pseudomonas aeruginosa*, which may be related to the humid environment surrounding the heel.

Our study has 2 main limitations. First, the sample size is limited, therefore the results should be interpreted cautiously, and more patients should be recruited in future studies to obtain more precise conclusions. Second, we did not analyze the risk factors of aseptic wound leakage and infection relapse because of the study’s retrospective design. To better identify potential risk factors, well-designed studies are necessary. However, this study provides a novel bone-preserving surgical strategy for CO treatment, with satisfying efficacy.

In conclusion, cortical bone windowing plus eggshell-like radical debridement with local implantation of antibiotic-loaded CS is an effective way to treat type III CO. However, patients receiving this treatment should be fully informed regarding the potential risk of aseptic wound leakage after local CS implantation. Future investigations may focus on the risk factors of aseptic wound leakage and infection relapse following local CS use as well as the efficacy of other substitute materials that could be implanted locally, such as bioactive glass.

NJ and XQZ contributed equally to this study. NJ, YJH, and BY designed the study. XQZ and QRL screened the medical record for patient inclusion. XQZ and LW collected the patient data. NJ and YJH conducted the data analysis. NJ and BY drafted the manuscript. All authors reviewed and revised the manuscript.

The authors are grateful for the funding support from the National Natural Science Foundation of China and also would like to thank Editage for language editing and revision.

Patient with a sharp puncture injury history, who presented with a discharging sinus tract in the lateral side of the calcaneus (a). The axial radiograph indicates infection spreading to the rear and medial sides (b, red arrows). Therefore, we selected a semi-ring incision parallel to the pelma. First, the sinus tract was excised and the surrounding soft tissues were debrided (c). Second, the devitalized cortical bone was windowed and removed to expose the calcaneal cancellous bone (d). Third, a curette was used to remove the infected cancellous bone and its surrounding osseous tissues, leaving a cavity in the residual calcaneus, similar to an eggshell (e). Fourth, vancomycin- and gentamicin-loaded CS was implanted into the cavity and surrounding the soft tissue (f). Finally, the skin was closed primarily (g). A postoperative radiograph showed the locally implanted CS (h). The patient recovered well at 1-year follow-up (i and j).
